# A non-invasive method for concurrent detection of multiple early-stage cancers in women

**DOI:** 10.1038/s41598-023-46553-7

**Published:** 2023-11-04

**Authors:** Ankur Gupta, Zaved Siddiqui, Ganga Sagar, Kanury V. S. Rao, Najmuddin Saquib

**Affiliations:** 1 PredOmix Health Sciences Private Limited, 10 Anson Road, #22-02 International Plaza, Singapore, 079903 Singapore; 2 PredOmix Technologies Private Limited, Tower B, SAS Tower, Medicity, Sector-38, Gurugram, 122002 India

**Keywords:** Cancer, Computational biology and bioinformatics, Systems biology, Biomarkers, Diseases, Oncology

## Abstract

Untargeted serum metabolomics was combined with machine learning-powered data analytics to develop a test for the concurrent detection of multiple cancers in women. A total of fifteen cancers were tested where the resulting metabolome data was sequentially analysed using two separate algorithms. The first algorithm successfully identified all the cancer-positive samples with an overall accuracy of > 99%. This result was particularly significant given that the samples tested were predominantly from early-stage cancers. Samples identified as cancer-positive were next analysed using a multi-class algorithm, which then enabled accurate discernment of the tissue of origin for the individual samples. Integration of serum metabolomics with appropriate data analytical tools, therefore, provides a powerful screening platform for early-stage cancers.

## Introduction

Cancer is rapidly emerging as the leading cause of premature death globally^1–3^. While development of more effective therapies is ongoing, early-stage detection of cancer offers a more viable strategy for reducing disease-related morbidity and mortality^4–9^. In addition to increasing the likelihood of treatment success, detection of cancer in its early stages also allows for improved quality of life, along with a significant reduction in the cost and complexity of treatment^10,11^. Multiple studies have shown that the 5-year survival rates are markedly higher in patients diagnosed with Stage I–II cancers as opposed to those who are diagnosed at Stage III-IV^12–17^. Unfortunately, however, screening tests are available only for a restricted set of cancers and these include breast, colorectal, cervical, lung, and prostate cancers^18–22^. While tests for these cancers have indeed contributed to reducing cancer-specific mortality^23,24^, their impact has remained sub-optimal because the efficacy of some of them remains questionable^25–28^. Furthermore, these screening approaches are designed to individually detect only a single cancer type^29^. Besides these five cancers, however, diagnosis for the remaining cancer types is still prompted by symptoms that appear only at the later stages of the disease.

Of the various approaches that are currently being marshalled to improve cancer diagnosis^30^, recent developments in the field of multi-cancer early detection (MCED) have shown promise^31–35^. MCED screening tests aim to capture signals from cell-free (cf)—or circulating tumour (ct)—DNA, or other circulating analytes shed by tumours into blood, that are associated with multiple cancers. Importantly, these tests also detect those cancers for which ‘standard of care’ screening modalities do not currently exist^28,32–35^. MCED tests are now being viewed as viable strategies for enhancing the depth and scope of cancer screening programs, thereby facilitating significant reductions in the cancer death rate. Although MCED tests currently under development do provide grounds for optimism, the fact that biomarker concentrations are low—which then have to be distinguished from the background noise of normal human physiology—have hampered efforts to achieve high detection sensitivity for early-stage cancers^28,35,36^. This poses a limitation because an effective screening test must have high detection sensitivity and specificity so that problems due either to under- or over-diagnosis are minimized^37,38^.

In a previous study we had adopted an alternate approach wherein we interrogated the serum metabolome for any modulations in metabolite patterns that correlated with the presence or absence of cancer^39^. Our rationale was derived from the fact that the metabolite composition of biological fluids reflects the health of an individual^40,41^. Furthermore, metabolome profiling appeared to be particularly relevant for cancer detection given that metabolic reprogramming is one of the key hallmarks of cancer cells^42–45^. Therefore we had reasoned that, by using appropriate data analysis tools, it should be possible to accurately extract metabolite ‘signatures’ that are characteristic of cancer. Our expectations were indeed borne out by the results obtained^39^. In that report we had shown, by combining untargeted serum metabolomics with machine learning-based data analysis, that we could detect Stage-0/I of the four female-specific cancers (breast, endometrial, cervical, and ovarian) with an average accuracy of around 98%^39^. Subsequently, in a follow-up study, we were also able to validate the performance of this test in a clinical setting (manuscript submitted).

The encouraging nature of these earlier results suggested to us that our approach could potentially be developed as an early-stage multi-cancer detection platform. To this end, we first sought to explore whether the scope of this method could be expanded to detect additional cancers in women, especially those in Stage-I of the disease. Results described here reveal that our methodology could indeed be readily adapted to concurrently detect the early stages of a total of 15 cancers in women with high accuracy. At a specificity of 99.3%, the detection accuracy of the individual cancers ranged from 94 to 100%, with an average sensitivity of > 99%. Furthermore, we were also able to successfully identify the ‘tissue of origin’ for the test samples at an overall accuracy of close to 92%.

## Results

### Details of samples included in the study

The demographic and clinical information of samples included in the study are presented in Table 1 and Supplementary Table 1. The age distribution of these samples ranged from 20 to 90 years. Nearly 95% of the samples came from individuals between the ages of 30 to 80 years, and the remaining 5% was split between individuals from the age groups of 20—30 years and 81–90 years (Supplementary Fig. 1). The majority of samples (92%) were from Caucasian with only 8% of samples coming from non-White who were either Hispanic, Asian, or African American women. The total number of cancer samples was 1926, which included samples from women with either breast, endometrial, cervical, ovarian, lung, AML, thyroid, melanoma, colorectal, kidney, NHL, pancreatic, head & neck, gastric, liver and bile duct cancers. Additionally, we also included 300 samples from healthy volunteers as the normal control subset.

**Table 1:** Description of the sample set employed in the study.

	Age (years)							BMI (kg/m^2^)		Ethnicity		Cancer stage				
	20–30	31–40	41–50	51–60	61–70	71–80	81–90	10 to 30	> 30	White	Non-white	0	I	II	III	IV
Normal control (n = 300)	19	50	98	83	40	10	0	166	11	203	97	0	0	0	0	0
Endometrial cancer (n = 304)	0	6	48	156	76	14	4	130	172	302	2	0	304	0	0	0
Breast cancer (n = 303)	2	41	80	111	53	14	2	270	24	246	57	36	267	0	0	0
Cervical cancer (n = 250)	20	79	76	48	21	6	0	90	22	233	17	70	180	0	0	0
Ovarian cancer (n = 262)	6	43	72	72	60	6	3	86	50	256	6	19	243	0	0	0
Lung cancer (n = 81)	0	2	16	48	13	2	0	30	12	80	1	7	72	0	0	2
Adult-acute myeloid leukemia (n = 71)	5	0	7	23	20	13	3	10	10	71	0	0	0	0	0	0
Thyroid cancer (n = 70)	8	12	22	21	6	1	0	23	12	70	0	0	67	3	0	0
Melanoma cancer (n = 86)	4	9	13	23	21	11	5	37	18	86	0	2	58	16	4	6
Colorectal cancer (n = 87)	1	7	12	22	25	16	4	74	13	87	0	0	62	19	5	1
Kidney cancer (n = 80)	1	4	8	22	40	5	0	49	30	80	0	0	69	5	4	2
Non-Hodgkin lymphoma (n = 50)	0	6	4	12	13	14	1	31	18	50	0	0	0	0	0	0
Pancreatic cancer (n = 75)	0	0	7	16	40	11	1	62	13	75	0	0	23	52	0	0
Liver & bile cancer (n = 34)	1	1	3	15	10	4	0	12	5	34	0	0	26	8	0	0
Gastric cancer (n = 85)	1	9	10	16	31	15	3	65	16	85	0	0	65	20	0	0
Head and neck cancer (n = 88)	8	5	5	28	29	10	3	48	14	88	0	14	48	26	0	0

Distribution of the sample numbers in terms of cancer type, age-groups, BMI, and ethnicity is given here. In addition, information on the cancer stage is also provided.

### Pre-processing of data prior to AI workflow

**Figure 1:**
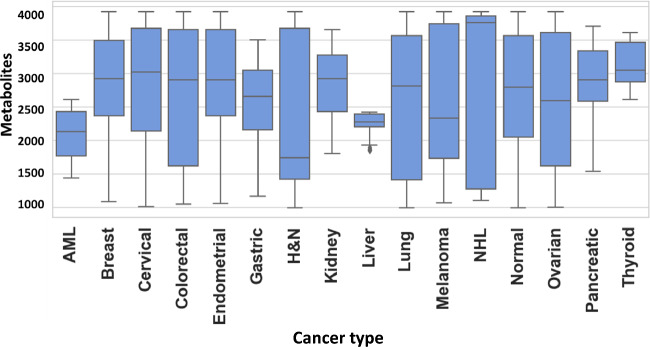
Distribution of metabolites detected across the individual cancer groups. Figure provides a graphical distribution of the number of metabolites in the individual samples of each of the 15 cancer types included in the study. The cumulative number of named metabolites for all samples within a given cancer type is given in the text.

**Figure 2:**
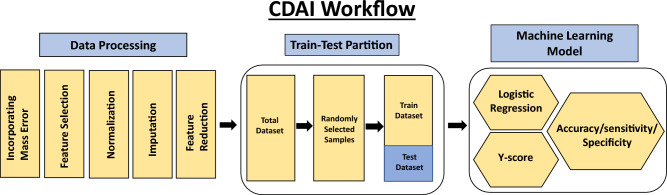
The data processing pipeline. The data processing approach used to develop the CDAI algorithm is illustrated here. Details are provided in the text.

An untargeted metabolomics workflow involving positive ion mode ultra-pressure liquid chromatography coupled to mass spectrometry (UPLC-MS/MS) was employed for the individual serum samples described in Table-1. This resulted in > 20,000 spectral features (RT, m/z pairs), which was then further resolved into known metabolites by using the Human Metabolome Database (HMDB). The number of known metabolites obtained by this process for the individual groups of normal control, breast cancer, endometrial cancer, cervical cancer, ovarian cancer, lung cancer, AML, thyroid cancer, melanoma, colorectal cancer, kidney cancer, NHL, pancreatic cancer, head & neck cancer, gastric cancer and liver & bile duct cancer were 2821, 3119, 3209, 3237, 2638, 2238, 2215, 2344, 2622, 2117, 1935, 2033, 2202, 2160, 2116, and 2045, respectively. The cumulative list across all the groups was found to comprise of 8312 unique metabolites, which were then used for further analysis. The distribution of these unique metabolites across the individual sample groups is shown in Fig. 1. We next processed this data through our in-house pipeline that included normalization, gap filling, data transformation, followed by feature filtering and selection (Methods, Fig. 2) to generate a matrix consisting of 5104 features representing the 1926 cancer samples, as well as the 300 normal control samples.

**Figure 3:**
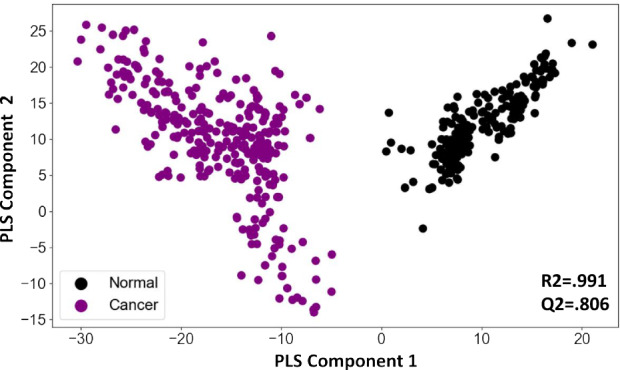
PLSDA plot distinguishes cancer samples from normal controls. Results of a partial least squares-discriminant analysis (PLS-DA) is shown here as a plot of the matrix of samples and ion boxes versus metabolite intensity. The explained variation parameter R-squared parameter for cancer vs normal (R2 = 0.991). Q-squared value was evaluated using a separate test for the PLSDA fit which was equal to(Q2 = 0.8065).

To determine whether the information contained in these features could distinguish between cancer samples and normal controls we first generated a PCA plot of cancer samples and normal controls with and without the QC samples. The initial plot was indicative for class separation (supplementary Fig. 2). Following this generated a PLSDA plot using the matrix. As shown in Fig. 3, the PLSDA plot could clearly differentiate cancer samples from normal control by segregating them into two distinct clusters (R2 = 0.991, Q2 = 0.806). To further develop this into a robust and sensitive method for cancer diagnosis, we resorted to AI analysis. The aim here was to more precisely capture variations in metabolite patterns that characterized the cancer samples on the one hand, and normal control samples on the other. In addition to cancer detection, we were also interested in developing an algorithm that enabled identification of the tissue of origin (TOO) in the case of cancer-positive samples. Accordingly then, we adopted a layered approach where we first focussed on accurately distinguishing cancer samples from the normal control, followed by the development of an algorithm for identifying the TOO of the cancer-positive samples.

### Cancer detection artificial intelligence (CDAI) algorithm for distinguishing cancer samples from normal controls

The first step was to develop an algorithm for the differentiation of cancer samples from normal controls. We termed this as the Cancer Detection Artificial Intelligence (CDAI) model. For this, the matrix data was randomly divided into training and test sets in comparable proportion between the individual cancers and the normal controls in order to cumulatively distinguish all 15 cancers listed in Table 1 from normal controls. A total of 150 normal control samples and 966 cancer samples were used as the training set, while the test set was comprised of 150 normal controls and 960 cancer samples (Table 2). The accuracy, sensitivity, and specificity values for the CDAI model were obtained by applying it to the training set and evaluating it on the test set (Table 2 and Fig. 2). To distinguish between cancer samples and normal control, the logistic regression function was applied to the training data.

$$ {\text{y}}\_{\text{score}} = {\text{x}}_{0} + {\text{x}}_{{1}} *{\text{I}}_{{1}} + {\text{ x}}_{{2}} *{\text{I}}_{{2}} + {\text{ x}}_{{3}} *{\text{I}}_{{3}} + \cdots \cdots \, + {\text{x}}_{{\text{n}}} *{\text{I}}_{{\text{n}}} $$

**Table 2:** Distribution of samples between the training and testing sets for development of the CDAI algorithm.

Sample type	Total sample number		Train		Test	
Normal	300	300 normal	150	150 normal	150	150 normal
Breast	303	1926 Cancer samples	152	966 Cancer samples	151	960 Cancer samples
Cervical	250		125		125	
Colorectal	87		44		43	
Endometrial	304		152		152	
Gastric	85		43		42	
H&N	88		44		44	
Kidney	80		40		40	
Liver	34		17		17	
Lung	81		41		40	
Melanoma	86		43		43	
NHL	50		25		25	
AML	71		36		35	
Ovarian	262		131		131	
Pancreatic	75		38		37	
Thyroid	70		35		35	

The partitioning of samples within each cancer group between the training and testing sets is given. Normal control samples were also similarly partitioned with 150 samples being taken for training and the remaining 150 samples being employed as the test subset.

Here, × 0 is a constant number, I_i_ (1 ≤ i ≤ n) is the intensity of metabolite i present in the respective sample. The total number of metabolites is represented by the symbol n(n ∈ [1000, 5104]). Supplementary Fig. 3 gives the value of coefficient x_i_(1 ≤ i ≤ n) for each metabolite.

**Figure 4:**
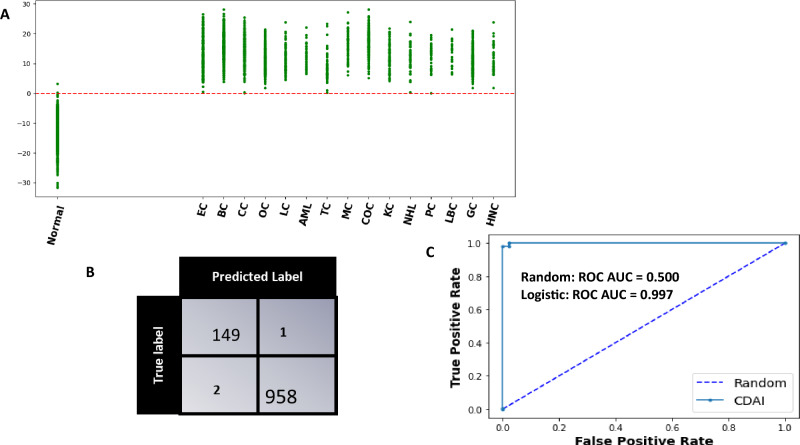
The CDAI model accurately differentiates cancer samples from normal controls. (**A**) The results obtained with the trained CDAI model for cancer versus normal control samples in the test set. A scatter plot of the y-scores obtained for the individual samples within each of the cancer types is shown. The y-score of each of the 15 cancers are shown separately. The resulting confusion matrix obtained on applying a threshold of a y-score of 0 is shown in (**B**) wherein the high cancer detection accuracy—independent of the cancer type—is clearly evident. The precision of the CDAI algorithm was also confirmed by the ROC-AUC curve shown in Panel C, where the Area under ROC_AUC curve was 0.997. Individual cancer types are abbreviated in (**A**) as follows: *BC* breast cancer, *EC* endometrial cancer, *CC* cervical cancer, *OC* ovarian cancer, *LC* lung cancer, *AML* acute myeloid leukaemia, *TC* thyroid cancer, *MC* melanoma cancer, *COC* colorectal cancer, *KC* kidney cancer, *NHL* non-Hodgkin’s lymphoma, *PC* pancreatic cancer, *HNC* head and neck cancer, *GC* gastric cancer, *LBC* liver and bile duct cancer.

The model was cross validated across 1000 random train-test split which yielded an average sensitivity, specificity of 99.6 (99.5–99.8), 99.3 (98.9–99.5) at 95 CI respectively. The evaluation of the trained model as applied on a single test set for a single partition of data is shown in Fig. 4. The scatter plot in panel A shows the Model Score for normal controls and cancer cases. It is evident that these scores are clearly different between normal controls and the samples derived from all the different cancer types being tested (Fig. 4A). Application of a threshold of 0 to differentiate between cancer samples and normal controls resulted in the confusion matrix shown in Fig. 4B. From the results depicted in this matrix, the overall cancer detection sensitivity calculated was 99.7% whereas the specificity was 99.3%. The ROC-AUC curve obtained for the CDAI model results is also shown in Fig. 4C. The sensitivity of our CDAI algorithm for correctly identifying samples within each cancer type as cancer-positive is given in Table 3. It is evident from the results shown in this table that, barring one sample from the cervical cancer subset and another from the thyroid cancer subset, all other samples were correctly identified as cancer-positive. These results confirm that our pipeline of untargeted serum metabolomics coupled with data analysis using our CDAI algorithm provides for cancer detection with very high sensitivity and specificity. Importantly, given that the majority of samples across all 15 cancers were either from Stage-0 or Stage-I of the disease, the results in Table 3 also underscore the particular utility of our method for early-stage cancer detection.

**Table 3:** Accurate identification of cancer-positive samples by the CDAI algorithm.

Cancer type	Total no. of samples tested	No. of samples correctly identified	Correct prediction (%)
Breast	151	151	100
Cervical	125	124	99.2
Colorectal	43	43	100
Endometrial	152	152	100
Gastric	42	42	100
H&N	44	44	100
Kidney	40	40	100
Liver	17	17	100
Lung	40	40	100
Melanoma	43	43	100
NHL	25	25	100
AML	35	35	100
Ovarian	131	131	100
Pancreatic	37	37	100
Thyroid	35	34	97.1
Normal	150	149	99.3

The number of samples from each cancer type that were tested are compared against those that were correctly identified as cancer-positive in each cancer type by the CDAI algorithm. Results obtained for the normal samples are also included where correct identification implies their discernment as cancer-negative samples.

### An artificial intelligence algorithm for determination of tissue of origin (TOOAI)

In the second step, our aim was to layer a multiclass AI model (tissue of origin, or, TOOAI model) on top of the CDAI model that would act on the cancer-positive samples from Table 3 to generate a multiclass score for each sample. That is, our aim was to score the relative probability with which the TOO of a given sample corresponded to each of the 15 cancer types that were being tested. Based on this grading then, it should be possible to identify the most likely TOO for that sample.

**Figure 5:**
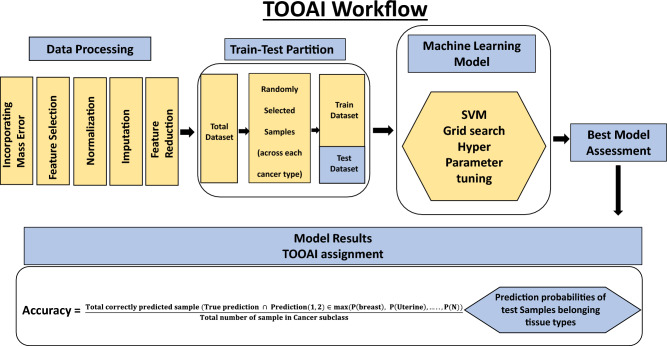
The workflow employed for development of the TOOAI algorithm. The workflow for the multi cancer detection TOOAI platform involves three major compartments common to the layer model. These include model building, assessment of the models, and determination of double class prediction accuracy.

Our cumulative set of 1926 cancer samples included those from endometrial cancer (n = 304), breast cancer (n = 303), cervical cancer (n = 250), ovarian cancer (n = 262), lung cancer (n = 81), leukemia (n = 71), thyroid cancer (n = 70), melanoma (n = 86), colorectal cancer (n = 87), kidney cancer (n = 80), lymphoma (n = 50), pancreatic cancer (n = 75), liver & bile duct cancer (n = 34), gastric cancer (n = 85), head & neck cancer (n = 88). The matrix data generated for these samples was randomly partitioned into training and test datasets in equal proportion as shown in Fig. 5 and Table 4. Then, a SVM multiclass classification model was made using the training samples to generate the TOOAI algorithm. The TOOAI algorithm was applied on those samples identified as cancer-positive by the CDAI algorithm, which generated 15 scores for each sample. Here, for a given samples, each score defined the probability of that sample belonging to one of the fifteen classes, or cancer types.

**Table 4:** Distribution of samples between the training and testing sets for development of the TOOAI algorithm.

Cancer type	Total sample number	Train		Test	
Breast	303	159	1064 Cancer samples	144	862 Cancer samples
Endometrial	304	159		145	
Cervical	250	133		117	
Ovarian	262	137		125	
Lung	81	30		51	
AML	71	18		53	
Thyroid	70	36		34	
Melanoma	86	61		25	
Colorectal	87	62		25	
Kidney	80	55		25	
NHL	50	31		19	
Pancreatic	75	50		25	
H&N	88	63		25	
Gastric	85	61		24	
Liver	34	9		25	

The partitioning of cancer-positive samples across the individual cancer groups into training and test sets is given. The total number of cancer samples employed for training was 1064, while that taken for testing was 862.

The multiclass classification TOOAI model was made using the training samples. The trained algorithm estimated tissue of origin probability of each of the sample, for each of the 15 cancer types, according to the formulae below:

$$\mathrm{P}(\mathrm{Endometrial})=\frac{1}{1+{e}^{y0+y1*I1+y2+I2+\cdots \cdots .}}$$

$$\mathrm{P}(\mathrm{Breast})=\frac{1}{1+{e}^{a0+y1*I1+y2+I2+\cdots \cdots .}}$$

$$\mathrm{P}(\mathrm{Cervical})=\frac{1}{1+{e}^{a1+y1*I1+y2+I2+\cdots \cdots .}}$$

$$\mathrm{P}(\mathrm{Ovarian})=\frac{1}{1+{e}^{a2+y1*I1+y2+I2+\cdots \cdots .}}$$

$$\mathrm{P}(\mathrm{Thyroid})=\frac{1}{1+{e}^{a3+y1*I1+y2+I2+\cdots \cdots .}}$$

$$\mathrm{P}(\mathrm{N})=\frac{1}{1+{e}^{an+y1*I1+y2+I2+\cdots \cdots .}}$$

Here, a_0_, a_1_, a_2_,…., a_n_ are constant number, I_i_ (1 ≤ i ≤ 8312) is the Normalized intensity of metabolite i present in the respective sample. N is number of cancer type classes included in the training set.

**Figure 6:**
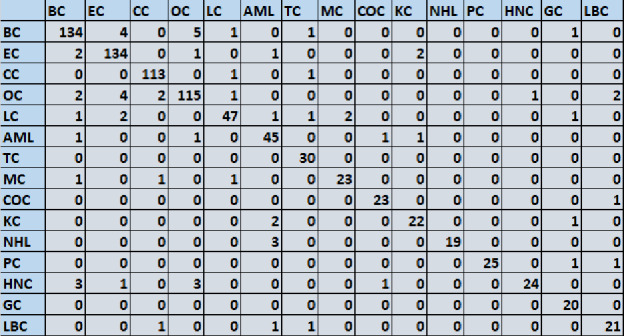
Performance of the TOOAI model. The confusion matrix built for discriminating between the 15 cancers, on the basis of a double class TOO prediction accuracy, by the TOOAI model is shown here. Details of model development are provided in Methods while an interpretation of the results is discussed in the text. Individual cancer types are abbreviated as follows: *BC* breast cancer, *EC* endometrial cancer, *CC* cervical cancer, *OC* ovarian cancer, *LC* lung cancer, *AML* acute myeloid leukaemia, *TC* thyroid cancer, *MC* melanoma cancer, *COC* colorectal cancer, *KC* kidney cancer, *NHL* non-Hodgkin’s lymphoma, *PC* pancreatic cancer, *HNC* head and neck cancer, *GC* gastric cancer, *LBC* liver and bile duct cancer.

The models were first assessed on the basis of their single class accuracy, wherein the first prediction (i.e. the highest probability score) was taken as the correct identification of the cancer TOO for a given sample. This analysis yielded an average accuracy across 15 cancers of 81% 95 CI (78.9–81.6) (results not shown). To further improve the accuracy, therefore, we considered a double-class prediction model in which the correct TOO likely occurred within the top two predictions from the model, calculated on the basis of the probability functions obtained as defined above. The double class prediction accuracies were evaluated for the test dataset and the confusion matrix for the final prediction is shown in Fig. 6. Double class prediction accuracy was obtained from the model by using the following formula:

$$\mathrm{Accuracy}= \frac{\mathrm{Total \, correctly \,  predicted  \, sample }(\mathrm{True  \, prediction }\cap \mathrm{ Prediction}(\mathrm{1,2})\in \mathrm{max}(\mathrm{P}\left(\mathrm{breast}\right),\mathrm{ P}\left(\mathrm{Uterine}\right),\dots ..,\mathrm{P}(\mathrm{N}))}{\mathrm{Total  \, number  \, of  \, sample  \, in  \, Cancer  \, subclass}}$$

Table 5 gives the results obtained for the double class prediction analysis. The significant improvement in prediction accuracy is evident here, which ranged from a low of 82% for gastric cancer to as high as 100% for Non-Hodgkin’s lymphoma and pancreatic cancer. Of the total of 862 cancer samples that were tested, TOO of 795 were correctly predicted resulting in an average accuracy of 92.2% (Table 5).

**Table 5:** Determination of the tissue of origin by the TOOAI algorithm.

Cancer type	# samples tested	# samples correctly classified	# samples incorrectly classified	Sensitivity
Breast	144	134	10	93.08
Endometrial	145	134	11	92.65
Cervical	117	113	4	96.64
Ovarian	125	115	10	91.60
Lung	51	47	4	93.02
AML (leukemia)	53	45	8	85.71
Thyroid	34	30	4	88.89
Melanoma	25	23	2	93.33
Colorectal	25	23	2	93.02
Kidney	25	22	3	88.10
NHL (lymphoma)	19	19	0	100
Pancreatic	25	25	0	100
Head & neck	25	24	1	94.12
Gastric	24	20	4	82.05
Liver & bile duct	25	21	4	83.33
Total	862	795	67	91.7 (average sensitivity)

Table lists the number of samples tested from each of the individual cancer type and compares this against that proportion of samples that were either correctly or incorrectly classified by the double class prediction of the TOOAI algorithm. The resulting sensitivity that was obtained for each cancer type is also included, along with the average sensitivity value across all the cancer types.

### Robustness of the CDAI

We also wanted to assess whether our method was subject to the vagaries of batch specific variability that is often seen in mass spectrometry data^46^. For this, we performed an experiment using a sample set that comprised of a pre-defined number of samples from each of the 15 cancers and normal controls as shown in Table 6 and Supplementary Table 3. This sample set was subsequently analysed over multiple times at intervals of 4–6 weeks, spanning a total period of 18 months. Analysis involved a UPLC-MS/MS run for the individual samples, followed by determination of the cancer-positive versus cancer-negative status with the CDAI algorithm. A total of ten such test runs were performed and the results, in terms of the CDAI accuracy, are shown in Table 6 and illustrated in Supplementary Fig. 4. Importantly, the coefficient of variation for the net sensitivity for cancer detection obtained across these ten test runs was as low as 0.003 (Supplementary Fig. 4), confirming the robustness of our overall methodology. We believe that this is a significant finding from the standpoint of further development of our approach as a possible MCED test.

**Table 6:** Robustness evaluation of the pipeline for cancer detection.

Cancer type	Batch runs									
	Run1	Run2	Run3	Run4	Run5	Run6	Run7	Run8	Run9	Run10
Breast	25/25: -100%	25/25: -100%	25/25: -100%	25/25: -100%	24/25: -96%	25/25: -100%	25/25: -100%	25/25: -100%	25/25: -100%	25/25: -100%
Endometrial	25/25: -100%	25/25: -100%	25/25: -100%	25/25: -100%	25/25: -100%	25/25: -100%	25/25: -100%	25/25: -100%	25/25: -100%	25/25: -100%
Cervical	23/23: -100%	23/23: -100%	23/23: -100%	23/23: -100%	23/23: -100%	23/23: -100%	23/23: -100%	23/23: -100%	23/23: -100%	23/23: -100%
Ovarian	25/25: -100%	25/25: -100%	25/25: -100%	25/25: -100%	25/25: -100%	25/25: -100%	25/25: -100%	25/25: -100%	25/25: -100%	25/25: -100%
Lung	24/24-100%	24/24-100%	24/24-100%	24/24-100%	24/24-100%	24/24-100%	24/24-100%	24/24-100%	24/24-100%	24/24-100%
AML (leukemia)	24/24-100%	24/24-100%	24/24-100%	24/24-100%	24/24-100%	24/24-100%	24/24-100%	24/24-100%	24/24-100%	24/24-100%
Thyroid	15/15-100%	15/15-100%	15/15-100%	15/15-100%	15/15-100%	15/15-100%	15/15-100%	15/15-100%	15/15-100%	15/15-100%
Melanoma	12/12-100%	12/12-100%	12/12-100%	12/12-100%	12/12-100%	12/12-100%	12/12-100%	12/12-100%	12/12-100%	12/12-100%
Colorectal	15/15-100%	15/15-100%	15/15-100%	15/15-100%	15/15-100%	15/15-100%	15/15-100%	15/15-100%	15/15-100%	15/15-100%
Kidney	12/12-100%	12/12-100%	12/12-100%	12/12-100%	12/12-100%	12/12-100%	12/12-100%	12/12-100%	12/12-100%	12/12-100%
NHL (lymphoma)	10/10-100%	10/10-100%	10/10-100%	10/10-100%	10/10-100%	10/10-100%	10/10-100%	10/10-100%	10/10-100%	10/10-100%
Pancreatic	12/12-100%	12/12-100%	12/12-100%	12/12-100%	12/12-100%	12/12-100%	12/12-100%	12/12-100%	12/12-100%	12/12-100%
Head & neck	15/15-100%	15/15-100%	15/15-100%	15/15-100%	15/15-100%	15/15-100%	15/15-100%	15/15-100%	15/15-100%	15/15-100%
Gastric	15/15-100%	15/15-100%	15/15-100%	15/15-100%	15/15-100%	15/15-100%	15/15-100%	15/15-100%	15/15-100%	15/15-100%
Liver & bile duct	15/15-100%	15/15-100%	15/15-100%	15/15-100%	15/15-100%	15/15-100%	15/15-100%	15/15-100%	15/15-100%	15/15-100%

While details of the experiment performed are described in the text, this table indicates the number of samples taken from each cancer type. Also included here is the cancer detection sensitivity obtained for each cancer type sample subset across all the ten runs. As shown in Supplementary Fig. 4, a comparison of net sensitivity yielded a CV of 0.003.

### Identification of features critical for cancer detection

Our matrix features were able to recognize named metabolites in the HMDB database. This renders our model results more amenable towards gaining useful insights into the metabolic adaptations that seemingly correlate with cancer development. To facilitate such future analysis, we sought to short-list those metabolites that contributed significantly to the cancer-specific signatures detected by the CDAI algorithm. For this we employed feature ranking, wherein weights of the CDAI model’s individual features—or named metabolites—involved in distinguishing between cancer and normal control samples were first sorted. Subsequently, these features were ranked using the recursive feature elimination technique, which involved elimination of one feature at a time. The top ranking metabolites that resulted from this exercise are listed in Supplementary Table 2.

## Discussion

Efforts to improve the detection of cancers at an early stage are currently being spurred by the development of MCED tests based on ctDNA analysis, which can detect multiple cancers with a single blood draw^47^. The attraction posed by such tests is that they facilitate detection of many additional cancers that would otherwise remain undetected until later stages, when prognosis is generally poor^48^. Mathematical modelling has suggested that inclusion of MCED tests to usual care can yield a significant positive effect in terms of substantially reducing the overall cancer mortality^49^. Nonetheless, despite the potential shown by ctDNA-based MCED tests, concerns have emerged that this approach may not represent a satisfactory proxy for biopsies of tumour tissues, especially for early-stage cancer detection. These concerns derive from the fact that the detection sensitivity and/or specificity obtained for early-stage cancers has generally been less than satisfactory^50,51^. While the low to negligible concentration of ctDNA present in the early stages of cancer has been identified as the primary cause, ctDNA variability based on type and status of the tumour is also another likely complicating factor^52–54^.

To circumvent the limitations inherent to ctDNA-based MCED tests, we had adopted an alternate approach that built on the widely accepted notion that metabolites serve better as proximal reporters of disease since their relative abundances are often directly related to pathogenic mechanisms^55–57^. Accordingly, we employed untargeted metabolomics—using high-resolution mass spectrometry—to generate a dense representation of the serum metabolome. The resulting data was then deconvoluted using a set of machine learning algorithms to first distinguish between cancer-positive and cancer-negative cases, followed by a further analysis of samples from the former group to identify the likely tissue of origin of the cancer. As demonstrated in our earlier report^39^, this approach was indeed unconstrained by the factors that limit accuracy of ctDNA-based MCED tests, at least for the cancers evaluated. An overall detection accuracy of as high as 98% was achieved for Stage-0/I of these cancers (^39^, manuscript submitted). However, since this previous study was restricted to detection of only the four female-specific cancers, we wanted to explore whether additional cancers could also be brought within the scope of this test. In this report we examined the ability of this method to detect a total of fifteen cancers in women, including the four previously evaluated female-specific cancers.

Results described here confirm that our approach of integrating untargeted metabolomics with machine learning-powered data analytics has strong potential for development as a high-fidelity screening test for early-stages of multiple cancers. This is evident from our finding that all 15 cancers tested could be detected with a sensitivity that ranged from 94 to 100%, at a specificity of 99.3%. Importantly, these cancers also included those that are known to be notoriously difficult to detect such as cancers of the pancreas, lung, kidney, ovary, liver, and sarcoma. Besides the fact that the early stages are largely asymptomatic, detection of these cancers is further hindered by their occurrence in tissues that are not readily accessible. The absence of any overt or specific symptoms in the early stages is also a characteristic of many of the remaining cancers in our list, as a result of which they too normally tend to go undetected for long periods of time^58–60^. Despite these inherent impediments, however, we were able to uniformly detect each of the individual cancers with high sensitivity and specificity. That is, our method facilitates concurrent detection of multiple cancers including those that are intractable to discernment by available screening modalities.

The fact that the samples employed for all cancer types were primarily derived from patients in Stage-I of the disease is another notable aspect of our study. As described in Table 1, while 31% of pancreatic cancer samples were from Stage-I, the proportion of early-stage cancers (Stage-0/I) was between 70 and 80% in the case of melanoma, colorectal, liver and bile, gastric, and head & neck cancers. For kidney cancer 86% of samples employed were derived from Stage-I, while this proportion was 96% for thyroid cancer and 98% (Stage-0/I) for lung cancer. For the remaining cancers, all samples tested were from Stage-0/I of the disease (see Table 1). Thus, given the preponderance of very early-stage cancer samples in our test set, results presented in this report go to further substantiate the unique capability of our method to accurately detect early-stages of at least the spectrum of cancers that were tested. This feature represents a significant advance given that early-stage detection has for long persisted as one of the principle challenges in the field of cancer diagnosis. Our earlier inference that a metabolomics-based approach is not confounded by limitations that plague ctDNA, circulating tumour cells, or protein biomarker-based strategies^39^, is also reinforced by these findings.

In addition to cancer detection, the inclusion of a multiclass algorithm (TOOAI model) in the data analysis pipeline also allowed us to predict the likely tissue of origin for those samples that proved to be cancer positive. For each test sample, the TOOAI model generated a list of 15 probability scores that defined the likelihood with which the tissue of origin of that sample corresponded to each of the cancer types tested. While an assignment of cancer type simply on the basis of the highest probability score yielded an overall accuracy of about 81%, this could be further enhanced to 92% by considering a double-class prediction where the tissue of origin was circumscribed to within the two most likely cancer types. Thus, tandem analysis of the serum metabolome using two separate algorithms enabled cancer detection to be coupled with localization of the tissue of origin. Complementing cancer detection with identification of the tissue of origin should aid in directing the subsequent tests required for diagnostic confirmation of the cancer.

Although it is an accepted truism that early diagnosis of cancer can save lives this goal has, nonetheless, proven elusive to date. However, results presented in both our previous^38^ and present study confirm that an interrogation of the serum metabolome, using a machine learning algorithm, for disease-specific metabolite signatures provides a fruitful strategy for detection of early-stage cancers with very high accuracy. Furthermore, by inclusion of a multiclass algorithm to further resolve the cancer-specific metabolite signatures, cancer detection could also be supplemented with tissue of origin identification. Thus, the approach described here clearly has potential for development as a multi-cancer screening test that is especially relevant for early-stage cancer detection and identification. We do acknowledge, however, that more rigorous clinical validation will be required before its potential can be translated into application in the field. Furthermore, the skewed distribution between the cancer and normal control samples in our sample set, as well as our adoption of a supervised approach for building the model, also demand a more rigorous assessment of the test robustness and reproducibility.

Another important question is the likely effect that comorbidities could have on the accuracy of our results. As shown in Supplementary Table 1, several of the donors for our sample set were those afflicted with other metabolism related diseases such as diabetes, heart disease, and hypertension among others (see Supplementary Table 1). The high cancer-detection accuracy that we, nonetheless, obtained suggests that at least these comorbidities do not exert a negative impact on the performance of our algorithm. This, however, does not rule out the possibility that there may be other classes of diseases (e.g. those related to inflammation, aging, etc.) that could affect the outcome. Studies are currently underway to address these diverse issues, and also evaluate the potential of our method as a multi-cancer screening test.

## Methods

### Sample details

A schematic of overall methodology is illustrated in Supplementary Fig. 5. A total of 1926 different cancer samples (Breast, Endometrial, Cervical, Ovarian, Lung, AML, Thyroid, Melanoma, Colorectal, Kidney, NHL, Pancreatic, Head & Neck, Gastric and Liver & bile duct) were taken to perform this study (Table 1, Supplementary Table 1). These samples were purchased from different biobanks such as Dx Biosamples (San Diego, CA), Reprocell USA Inc. (Beltsville, MD), and Fidelis Research AD (Beltsville, MD) (Sofia, Bulgaria). Samples including both cancer and healthy controls were from these three biobanks. The distribution of total number of cancer samples among these biobanks were 742 from Dx Biosciences, 807 from Reprocell USA and 377 from Fidelis Research AD. While, 150 Normal samples from Dx Biosciences, 100 from Reprocell USA and 50 from Fidelis Research AD. Samples obtained were categorically from treatment naïve women patients in various stages of the individual cancers (Table 1). Clinical information of the samples that included histological stage and grade, along with cancer’s TNM classification of the cancer.

### Sample indexing

A unique identification number was used to index the samples. To correctly assign samples for extraction and relative registering of result output, this number was used. This number was used to track and recall each sample with derived aliquots. Samples were kept at −80 °C until they were processed.

### Extraction of metabolites from serum samples

Extraction of metabolites from serum was done as previously described^39^. Briefly, serum samples were thawed on ice and then mixed prior to extraction. For metabolite extraction, 10 µl of serum was aliquoted into a 1.5 ml microcentrifuge tube (Genaxy, Cat No. GEN-MT-150-C. S). To this, 30 µl of chilled methanol (Merck, Cat. No. 1.06018.1000) was added and briefly vortexed. This mixture was then kept at −20 °C for 60 min.

After the incubation, the sample was centrifuged (Sorvall Legend Micro17, Thermo Fisher Scientific, Cat. No. Ligend Micro 17) at 10,000 rpm for 10 min. Supernatant (27 µl) was then carefully aspirated into a fresh microfuge tube without disturbing the pellet. Speed vacuum (ThermoFisher Scientific, Cat. No. SPD1030-230) was employed at low energy for 30 min to dry the supernatant. This dried sample was either stored at −80 °C for later use or reconstituted immediately with 30 µl methanol: water (1:1) for LCMS injection.

### Liquid chromatography and mass spectrometric (LC–MS/MS) analysis of serum metabolites

An untargeted metabolomics approach was adopted for this study^39^. In this method, a scan range (66.7–1000 Da) was typically selected to capture the metabolite pattern in the sample. A Dionex LC system (Ultimate 3000) coupled with a QExactive (Thermo Scientific) mass spectrometer was employed for this analysis. Samples were analyzed using positive polarity in ESI ionization, after injecting 10 µl of sample onto an Acquity UPLC HSS T3 column (Waters, 1.8 micron, 2.1 × 100 mm, Part No. 186003539). The working temperature for this stationary phase was 37 °C. Mobile phase A (water + 0.1% formic acid) and mobile phase B (methanol + 0.1% formic acid) was used for the gradient where the total run time was14 minutes. The gradient was initially held constant for one minute at 5% B. Then, was increased to 95% B in 7 min and was held for another 2 min at 95% B. Finally, gradient returned to 5% B by 14 min. The eluent was connected online to the QExactive source for ionization using 4 kV of voltage. The mass spectrometer was calibrated with the vendor recommended schedule to maintain the mass accuracy of 5 ppm. Optimized resolution for sample run was 70,000 with an AGC target of 1e6.

### Maintenance of mass spectrometric data quality

Mass spectrometry data variation was reduced by combining several controls with the experimental samples. Instrument performance and chromatographic alignment over the time was maintained using the QC samples. Additionally, this QC also served as a technical replicate throughout the study. A blank gradient run was incorporated after each sample injection to reduce the carryover problem associated with the stationary phase.

### Pre-processing of mass spectrometry data prior to AI workflow

The mass spectrometry data produced frequently varies between batches. We sought to control this variation by incorporating a set of pre-processing steps before applying the AI workflow. While a schematic of the overall process is depicted in Figs. 2 and 5, the individual steps involved were as follows.

1. *Inclusion of mass error in the data*: Despite using very rigorous procedure to avoid possible variation in data, mass errors are prevalent in metabolomics data. This error results in slightly different masses for the same metabolite in two samples. This posed a challenge to compare the intensity of the same metabolite across samples. This step of intensity comparison is essential to form patterns that are required in AI data analysis. As previously described^39^, an approach of adaptive virtual lock mass (VLM) was used to counter such variations. In principle, this approach relies on the fact that mass errors increase with increasing mass. We adapted this approach with our dataset and combined parts per million (ppm) mass errors with the metabolite identified by HMDB database. VLM boxes were created in alignment with the masses of metabolites identified by HMDB database and searched across the batches. The outcome of this exercise was an initial matrix of 8312 metabolites or features. Further, this matrix was trimmed with the removal of both plant or plant-derived, and drug or drug-derived metabolites. This resulted in a refined matrix of 5104 metabolites or features.
2. *Data filtering*: The presence of noise in a data set can increase the model complexity and time of learning, which degrades the performance of learning algorithms. Data filtering is a process of noise reduction as well as dimensionality reduction by which an initial set of raw data contains target specific attributes and is reduced to a more manageable data format.
3. *Data normalization/standardization*: Normalization techniques are required to reduce the variations in the data since the metabolic data fluctuate under different mass spectrometer parameters. Different normalization methods were tried such as Quantile Normalization, Variance Stabilization Normalization, Best Normalization, Probabilistic Quotient Normalization.
  Data standardization is a data processing workflow that converts the structure of different datasets into one common format of data. It deals with the transformation of datasets after the data is collected from different sources and before it is loaded into target systems. Various data standardization methods like standard normalization, L1 and L2 norm standardization were employed in the data set.
  A combination of Standardization and Normalization was used for the two-tiered algorithm. We found Quantile normalization was best suited for CDAI based on the accuracy in the training set and across the validation batches. This method was further adapted to our datasets to enable the normalization of new samples with respect to training datasets and testing one sample at a time. For TOOAI the raw data was first transformed using log base 10, and then subjected to Quantile normalization followed by standard scaler standardization.
4. *Missing value imputation*: It is well established that missing values in untargeted metabolomics data can be troublesome. In large metabolite panels, measurement values are frequently missing and, if neglected or sub-optimally imputed, can cause biased study results. Various supervised and unsupervised multiple imputation techniques like Iterative Imputer, missforest, simple impute, KNN impute were employed and the effects of sample size, percentage missing, and correlation structure on the accuracy of the imputation methods were evaluated. Finally, KNN imputation (n_neighbours = 5) was chosen out to be the most appropriate for our dataset. For CDAI we imputed the whole dataset uniformly. However, we followed selective imputation for TOOAI algorithm, where we selectively imputed 15 cancer classes in the training set, but the test set was kept non imputed.
5. *Feature reduction*: Dimensionality reduction is the process of reducing the number of random variables under consideration, by obtaining a set of principal variables. This is a critical step in high dimensional data as it takes care of curse of dimensionality, multi-collinearity, noise, computational cost, and visualization. Feature Extraction can be unsupervised (PCA) or supervised (LDA, PLS-DA etc.). Various Feature reduction techniques were evaluated based on data variance capture and class separation namely PLSDA R2 maximization, RFE, PCA, Non-negative Matrix Factorization, LDA. These were evaluated on the basis of their effect on overall accuracy in CDAI classification and, finally, PLSDA was used for feature reduction in CDAI as well as for the TOOAI.
6. *Machine learning model development*: After completing the above pipeline, the data was then fed into the AI machinery. AI models were made to differentiate cancers from normal and then between the individual cancers.

Keeping in mind the potential clinical applications of our data analysis pipeline, a tiered approach was used here in which an AI model was first developed for cancer signal detection (the CDAI Model). Following this, the TOOAI Model was developed to classify the tissue of origin for the cancer positive sample. The total 2226 samples taken for this study included endometrial cancer (n = 304), breast cancer (n = 303), cervical cancer (n = 250), ovarian cancer (n = 262), lung cancer (n = 81), leukemia (n = 71), thyroid cancer (n = 70), melanoma (n = 86), colorectal cancer (n = 87), kidney cancer (n = 80), lymphoma (n = 50), pancreatic cancer (n = 75), liver & bile duct cancer (n = 34), gastric cancer (n = 85), head & neck cancer (n = 88), and 300 normal control samples. The matrix produced from the data generated from these samples was then utilized for further analysis as described in “Results”.

### Development of the algorithm for the CDAI model

Of the total of 2226 samples, 1926 samples were from the 15 cancer classes and 300 were normal controls. Normal controls were samples from volunteers who had no cancer. The data was randomly partitioned into training and test datasets in equal proportion. This resulted in 966 Cancer samples and 150 Normal Controls in training set, and 960 Cancer samples and 150 Normal Controls in test set (Table 2). A complete schematic of the steps for cancer detection is shown in Fig. 2 and the model was evaluated using parameters log loss, Accuracy, Sensitivity, Specificity.

Parametric machine learning models were applied on the training data to obtain a score function depending on the intensity values of the features. The Class balancing parameters were configured in the model to deal with the imbalance of cancer and the control samples in the training dataset. The final trained model generated a score of each sample by using the following formulae:

$$ {\text{y}}\_{\text{score}} = {\text{x}}_{0} + {\text{x}}_{{1}} *{\text{I}}_{{1}} + {\text{ x}}_{{2}} *{\text{I}}_{{2}} + {\text{ x}}_{{3}} *{\text{I}}_{{3}} + \cdots \cdots \, + {\text{x}}_{{\text{n}}} *{\text{I}}_{{\text{n}}} $$

Here, × 0 is a constant number, I_i_ (1 ≤ i ≤ n) is the intensity of metabolite i present in the respective sample. The total number of metabolites is represented by the symbol n(n ∈ [1000,5104]). Supplementary Fig. 3 gives the value of coefficient x_i_(1 ≤ i ≤ n) for each metabolite.

The model was cross validated using 1000 random train test split and the average sensitivity, specificity, and accuracy at 95 CI was obtained. The y score plot of the trained model as applied on test set for a single partition of data containing 15 cancer classes and normal control is shown in Fig. 4. The scatter plot shows the Model Score for Controls and Cancer cases. The ROC-AUC probability curve showed a high degree of separability between the cancer and the normal controls. The model scores are clearly seen to be different between Controls and Cancer samples where on applying a threshold of y-score of zero to differentiate between two types of results in a confusion matrix as shown. Sensitivity, Specificity, and Accuracy can be calculated from the below formulae:

$$\mathrm{Accuracy}: \frac{TP+TN}{TP+TN+FP+FN}$$

$$\mathrm{Sensitivity}: \frac{TP}{TP+FN}$$

$$\mathrm{Specificity}: \frac{TN}{TN+FP}$$

**Table:** 

	Predicted		
		Negative	Positive
Actual	Negative	True negative (TN)	False positive (FP)
	Positive	False negative (FN)	True positive (TP)

### Development of the algorithm for the TOOAI model

In brief, the TOOAI model is a multiclass algorithm that evaluates the probability score for each cancer positive sample, which defines the tissue from which the cancer positive signal has originated. For developing this algorithm the dataset containing the cancer samples was first processed according to the steps explained in the earlier section. Here, out of total 1926 Cancer samples, samples were Endometrial Cancer, Breast Cancer, Cervical Cancer, Ovarian Cancer, Lung Cancer, Kidney Cancer, Thyroid cancer, Acute myeloid lymphoma, non-Hodgkin’s lymphoma, Pancreatic cancer, Colorectal cancer, Liver cancer, Gastric cancer, Melanoma cancer, head & neck cancer (Table 1). The data was randomly partitioned into training and test datasets in equal proportion and complete distribution of training and testing distribution in this layer is shown in Table 4.

The Machine learning environment was set for python 3.10.4. Various algorithms for example Support Vector Machine (SVM), Logistic one versus rest (LOVR), Stochastic gradient descent (SGD) algorithms etc. were evaluated in order to ascertain the best possible model for cancer type identification. The optimal set of hyperparameters for these models were obtained using exhaustive training testing by python Grid search CV package.

This Predict probability output of these models resulted in `15 probability scores for each sample, with each score defining probability of the respective sample belonging to one of the 15 cancer tissue types. The models were assessed based on their single class prediction accuracy and the best model was chosen for further evaluation. Out of these SVM gave the best test accuracy which was further cross validated using 100 random train-test split of the data. The trained algorithm finds tissue of origin probability for each of the sample according to the formulae below:

$$\mathrm{P}(\mathrm{Endometrial})=\frac{1}{1+{e}^{y0+y1*I1+y2+I2+\cdots \cdots .}}$$

$$\mathrm{P}(\mathrm{Breast})=\frac{1}{1+{e}^{a0+y1*I1+y2+I2+\cdots \cdots .}}$$

$$\mathrm{P}(\mathrm{Cervical})=\frac{1}{1+{e}^{a1+y1*I1+y2+I2+\cdots \cdots .}}$$

$$\mathrm{P}(\mathrm{Ovarian})=\frac{1}{1+{e}^{a2+y1*I1+y2+I2+\cdots \cdots .}}$$

$$\mathrm{P}(\mathrm{Thyroid})=\frac{1}{1+{e}^{a3+y1*I1+y2+I2+\cdots \cdots .}}$$

$$\mathrm{P}(\mathrm{N})=\frac{1}{1+{e}^{an+y1*I1+y2+I2+\cdots \cdots .}}$$

Here, a_0_, a_1_, a_2_,…., a_n_ are constant number, I_i_ (1 ≤ i ≤ 5104) is the Normalized intensity of metabolite i present in the respective sample. N is the number of cancer type classes included in the training set.

Using the scores for each class obtained we defined a double class prediction accuracy of the model, here the double class prediction accuracy will mean an occurrence of correct prediction in the top two predictions from the model using the above defined probability function.

The double class prediction accuracies were evaluated for the single test dataset as an example and the confusion matrix for the final prediction are shown in Fig. 6. Table 5 shows double class prediction accuracy for the same. The prediction accuracy for the double class prediction from the model were evaluated using the following formulae:

$$\mathrm{Accuracy}= \frac{\mathrm{Total  \, correctly  \, predicted  \, sample }(\mathrm{True  \, prediction }\cap \mathrm{ Prediction}(\mathrm{1,2})\in \mathrm{max}(\mathrm{P}\left(\mathrm{breast}\right),\mathrm{ P}\left(\mathrm{Uterine}\right),\dots ..,\mathrm{P}(\mathrm{N}))}{\mathrm{Total  \, number \,  of \,  sample \,  in \,  Cancer  \,  subclass}}$$

### Supplementary Information

Supplementary Figures.

Supplementary Table S1.

Supplementary Table S2.

Supplementary Table S3.

## Data Availability

The datasets used and/or analysed during the current study available from the corresponding author on reasonable request.
