# Supplementary material for: A non-invasive method for concurrent detection of multiple early-stage cancers in women
Source: Sci Rep. 2023 Nov 4;13:19083. doi: 10.1038/s41598-023-46553-7 (PMC10625604; doi:10.1038/s41598-023-46553-7)

**A NON-INVASIVE METHOD FOR CONCURRENT DETECTION OF MULTIPLE EARLY-STAGE CANCERS IN WOMEN**

**Ankur Gupta^1,2^, Zaved Siddiqui^1,2^, Ganga Sagar^2^, Kanury V.S. Rao^1,2^ & Najmuddin Saquib^1,2, *^**

Supplementary Information

**Supplementary Figure 1:** This figure depicts age-wise distribution of samples among healthy and cancer individuals. A total number of 1926 cancer serum samples of 15 mentioned cancers were collected with an additional 300 samples representing the normal control set. The box plot shows the age distribution of the cancer and the normal control samples distributed across (>18 to <=90) years used for the current study.

**Supplementary Figure 2:** The PCA plot for the metabolite intensities for cancer samples and normal controls. A show the PCA plot for normal and cancer controls, B is plotted along with the positive and negative control samples.

**Supplementary Figure 3:** The coefficient/weights of each metabolite involved in contributing to the CDAI-extracted signature for differentiating cancer samples from the normal controls is depicted here.

**Supplementary Figure 4:** Evaluation of the robustness of our method for cancer detection. While details of this experiment are provided in the manuscript text, the coefficient of variation (CV) was calculated from the y-scores obtained for the individual samples in each cancer group. A CV value of 1e-3 was obtained.

**Supplementary Figure 5:** A schematic representation of our pipeline integrating UPLC-MS/MS based serum metabolome analysis with AI algorithms for cancer detection (CDAI) and tissue of origin determination (TOOAI).


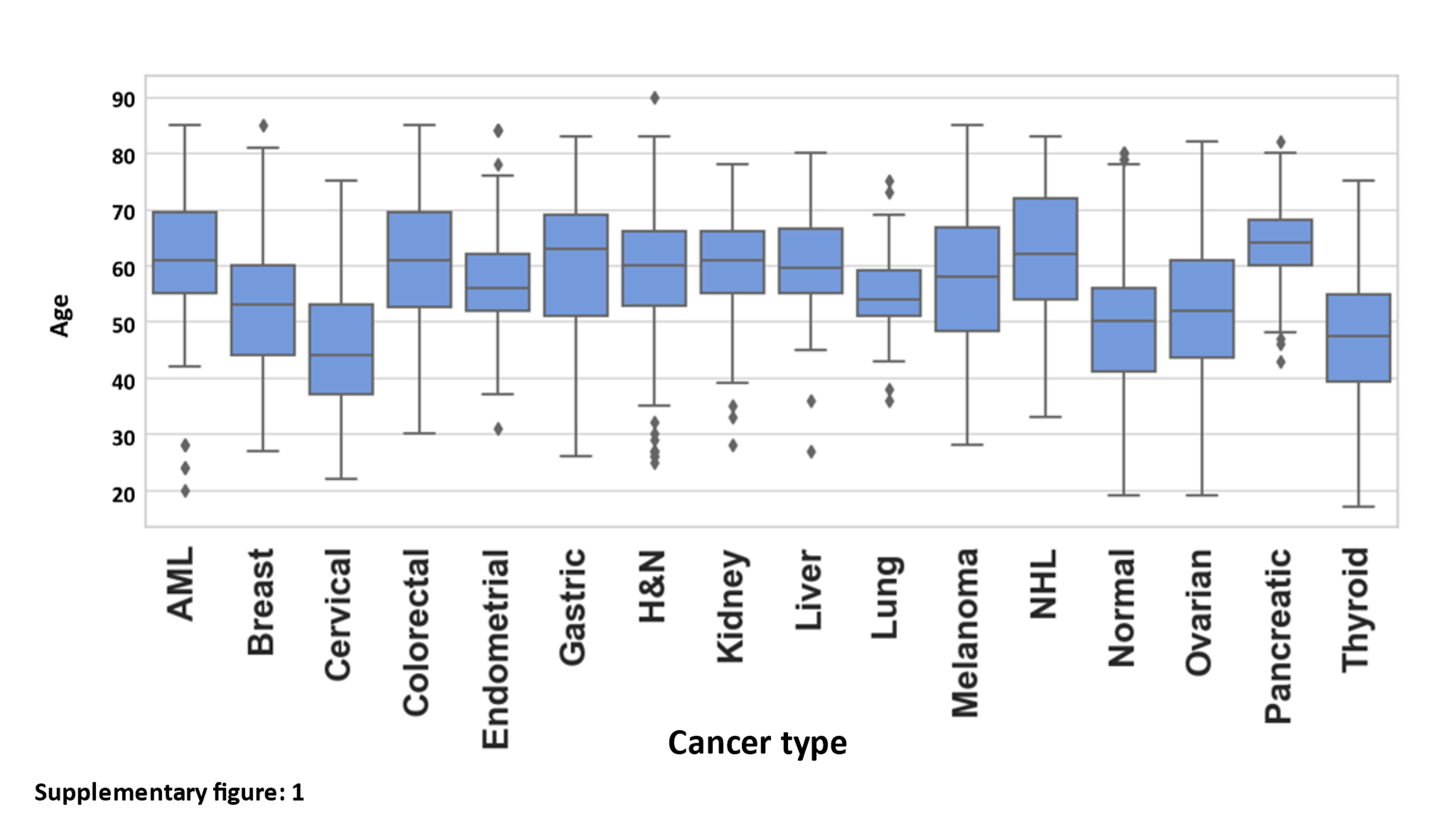


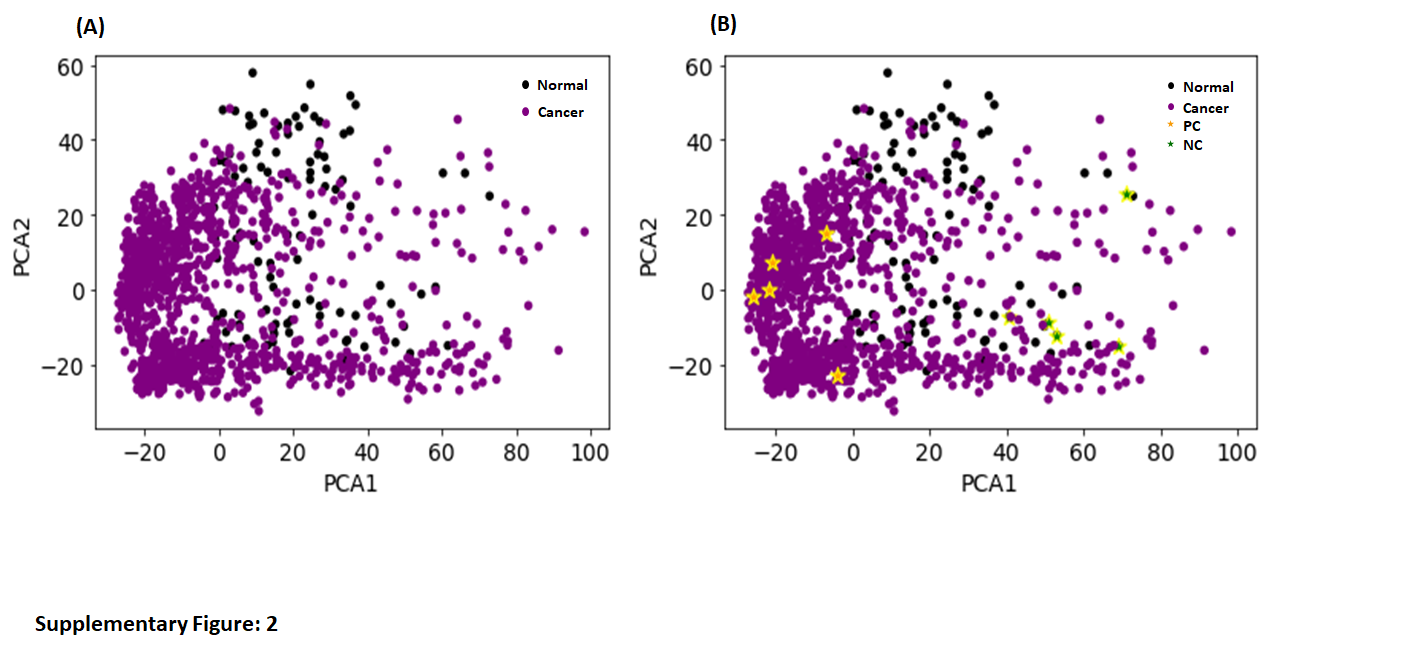


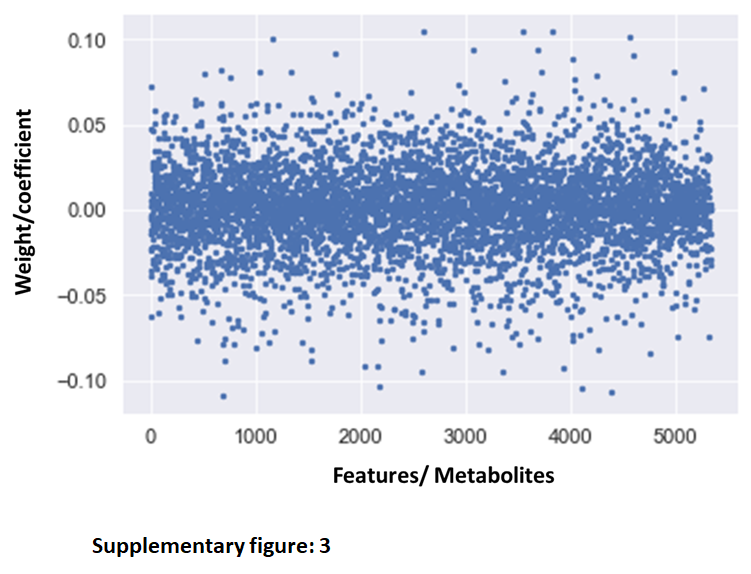

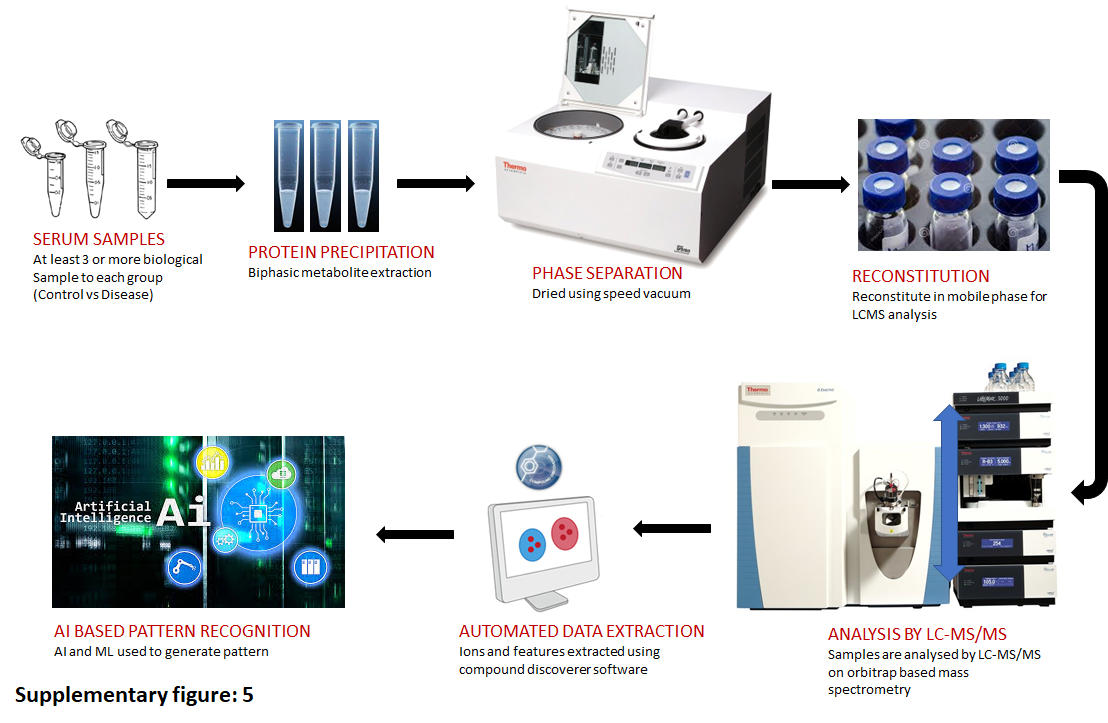

Supplement: Supplementary file 1 — Supplementary Figures. [file 41598_2023_46553_MOESM1_ESM.docx]
