# Supplementary material for: A non-invasive method for concurrent detection of multiple early-stage cancers in women
Source: Sci Rep. 2023 Nov 4;13:19083. doi: 10.1038/s41598-023-46553-7 (PMC10625604; doi:10.1038/s41598-023-46553-7)
Supplement: Supplementary file 4 — Supplementary Table S3. [file 41598_2023_46553_MOESM4_ESM.docx]

**Supplementary Table 3.** Details of the sample-set employed for assessing the robustness of the test protocol and CDAI algorithm.

| **Sample Type** | **Number** |
| --- | --- |
| Breast Cancer | 25 |
| Endometrial Cancer | 25 |
| Cervical Cancer | 23 |
| Ovarian Cancer | 25 |
| Lung Cancer | 24 |
| AML | 24 |
| Thyroid Cancer | 15 |
| Melanoma | 12 |
| Colorectal Cancer | 15 |
| Kidney Cancer | 12 |
| NHL | 10 |
| Pancreatic Cancer | 12 |
| Head & Neck Cancer | 15 |
| Gastric Cancer | 15 |
| Liver & Bile Duct Cancer | 15 |
| Normal Controls | 25 |
| **Total** | **292** |
